# Development of a novel angiotensin converting enzyme 2 stimulator with broad implications in SARS-CoV2 and type 1 diabetes

**DOI:** 10.21203/rs.3.rs-2642181/v1

**Published:** 2023-04-05

**Authors:** Niwanthi Rajapakse, Haru Nomura, Melanie Wu, Jiangning Song, Andrew Hung, Shirley Tran, Hang TA, Fahima Akther, Yuao Wu, Matt Johansen, Keng Chew, Vinod Kumar, Trent Woodruff, Richard Clark, Johannes Koehbach, Bruno Lomonte, Carlos Rosado, Merlin Thomas, Marion Boudes, Cyril Reboul, Lachlan Rash, Linda Gallo, Sumia Essid, Dominika Elmlund, Stefan Miemczyk, Nicole Hansbro, Bernadette Saunders, Warwick Britton, Peter Sly, Ayaho Yamamoto, Julian Fernandez, Peter Moyle, Kirsty Short, Philip Hansbro, Sanjaya Kuruppu, Ian Smith

**Affiliations:** The University of Queensland; The University of Queensland; School of Chemistry and Molecular Biosciences, The University of Queensland; Monash University; School of Science, RMIT University; School of Biomedical Sciences, The University of Queensland; Griffith University; Griffith University; Griffith University; School of Life Sciences, University of Technology; School of Chemistry and Molecular Biosciences, The University of Queensland; School of Biomedical Sciences, The University of Queensland; University of Queensland; The University of Queensland; School of Biomedical Sciences, The University of Queensland; Instituto Clodomiro Picado; Monash University Clayton Campus; Department of Diabetes, Central Clinical School, Monash University; Monash University; Monash University; The University of Queensland St Lucia QLD 4072, Australia; School of Biomedical Sciences, The University of Queensland; School of Biomedical Sciences, The University of Queensland; Monash Biomedicine Discovery Institute, Monash University; University of Technology Sydney; University of Technology Sydney; School of Life Sciences, University of Technology; Centenary Institute, The University of Sydney; The University of Queensland; Instituto Clodomiro Picado; The University of Queensland; University of Queensland; Centenary Institute and University of Technology Sydney; Monash University; Monash University

## Abstract

Angiotensin-converting enzyme 2 (ACE2) is protective in cardiovascular disease, lung injury and diabetes yet paradoxically underlies our susceptibility to SARs-CoV2 infection and the fatal heart and lung disease it can induce. Furthermore, diabetic patients have chronic, systemic inflammation and altered ACE2 expression resulting in increased risk of severe COVID-19 and the associated mortality. A drug that could increase ACE2 activity and inhibit cellular uptake of severe acute respiratory syndrome coronavirus 2 (SARs-CoV2), thus decrease infection, would be of high relevance to cardiovascular disease, diabetes and SARs-CoV2 infection. While the need for such a drug lead was highlighted over a decade ago receiving over 600 citations,^1^ to date, no such drugs are available.^2^ Here, we report the development of a novel ACE2 stimulator, designated ‘2A’(international PCT filed), which is a 10 amino acid peptide derived from a snake venom, and demonstrate its in vitro and in vivo efficacy against SARs-CoV2 infection and associated lung inflammation. Peptide 2A also provides remarkable protection against glycaemic dysregulation, weight loss and disease severity in a mouse model of type 1 diabetes. No untoward effects of 2A were observed in these pre-clinical models suggesting its strong clinical translation potential.

## Results

### Discovery of 2A.

**Figure 1:**
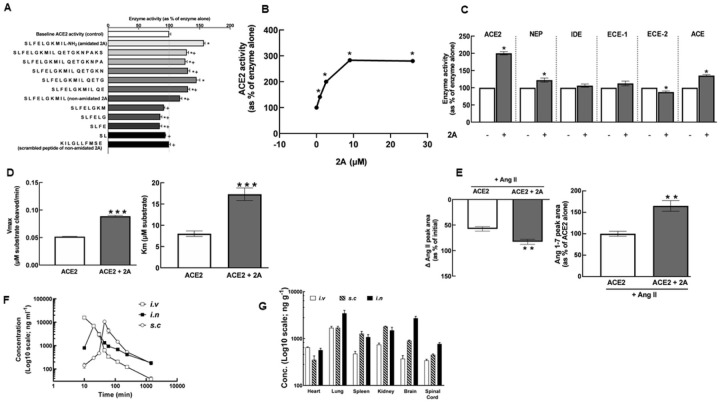
2A increases recombinant human ACE2 activity *in vitro and changes the conformation of ACE2*. (A) The effects of the truncated analogues of 20 AA parent peptide of 2A (2.6 μM) on ACE2 activity (n=4–5); *significantly different compared to ACE2 alone, and ^+^significantly different compared to ACE2 + amidated 2A; one-way ANOVA followed by Tukey’s post-hoc test; *P* < 0.05. (B) The effects of increasing concentrations of 2A (0.9–26 μM) on ACE2 activity (n=10; SEM are too small to be displayed); *significantly different compared to ACE2 alone; one-way ANOVA followed by Tukey’s post-hoc test; *P* < 0.001. (C) The effects of 2A (2.6 μM) on the enzyme activity of Zn^2+^ metalloproteases closely related to ACE2 (n=5–10); *significantly different compared with respective enzyme alone; unpaired *t*-test; *P* < 0.01. (D) The effects of 2A (2.6 μM) on V_max_ and K_m_ of ACE2 (n=4–5); ***significantly different compared with enzyme alone; unpaired *t*-test, *P* < 0.001. (E) The effects of 2A (1.7 μM) on breakdown of the natural substrate of ACE2, Ang II and formation of Ang 1–7 (n=4); **significantly different compared with enzyme alone, unpaired *t*-test, *P* < 0.01. (F-G) 2A (1mg/kg) was administered to normal 12-week-old C57Bl/6J mice via intravenous (i.v; n=5), intranasal (i.n; n=5) and subcutaneous (s.c; n=5) routes. LCMS was then used to determine plasma profile over 24 h (F) and tissue distribution of 2A, 24 h post administration (G). Data represent mean ± SEM. Abbreviations: ACE2, angiotensin converting enzyme 2; NEP, neprilysin; IDE, insulin degrading enzyme; ECE-1, endothelin converting enzyme-1; ECE-2, endothelin converting enzyme-2; ACE-1, angiotensin converting enzyme-1.

We identified a 20 amino acid peptide from the sequence of a myotoxin in *Bothrops asper* venom which stimulates recombinant human ACE2 enzyme activity^3^. C-terminal truncations indicated that the first 10 amino acids represent the minimum optimum sequence required for ACE2 stimulation (Figure 1A). ACE2 activity was not significantly different in the presence of the scrambled version of the 10 amino acid peptide (2.6 μM; n=5, Figure 1A). Alanine substitutions at positions 2, 5, 7 and 10 of the sequence of 2A significantly reduced ACE2 stimulation compared with native 2A (Supplementary figure 1A), indicating the importance of these residues for ACE2 stimulation.

#### Stability in cell culture

C-terminally amidated and non-amidated versions of 2A were added to HEK293 cells grown in culture, and their breakdown was monitored by LCMS at time 0 min (T_0 min_) and 60 min (T_60 min_). The amount of C-terminally amidated 2A remaining in the medium of HEK293 cells (54 ± 4% at T_60 min_ compared with T_0 min_) was significantly greater compared with non-amidated 2A (29 ± 3% at T_60min_ compared with T_0 min_; P<0.05; Supplementary figure 1B). We found only 63 ± 14% of C-terminally amidated 2A remained in the cell culture medium in the absence of cells at T_60 min_ indicating possible adherence of 2A to plastic (Supplementary figure 1B). We used the C-terminally amidated 2A in all subsequent experiments.

### 2A increased recombinant human ACE2 catalytic activity

2A increased recombinant human ACE2 activity in a concentration dependent manner (Figure 1B). A submaximal concentration of 2.6 μM was chosen for subsequent assays using quenched fluorescent substrate (QFS). 2A also increased the activity of closely related enzymes neprilysin (NEP) and angiotensin converting enzyme (ACE) but to a much lesser extent and significantly decreased the activity of endothelin converting enzyme-2 (ECE-2; Figure 1C). In the presence of 2A, the V_max_ of ACE2 (0.090 ± 0.002 μmols of substrate cleaved/min) was significantly higher compared with ACE2 alone (0.050 ± 0.001 μmols of substrate cleaved/min; *P* < 0.001; Figure 1D), providing evidence for enhanced catalytic activity. In the presence of 2A, the K_m_ of ACE2 (17.30 ± 1.50 μM) was significantly higher compared with ACE2 alone (8.03 ± 0.65 μM; *P*<0.001; Figure 1D).

ACE2-dependent conversion of natural substrate Ang II to Ang 1–7 was monitored using liquid chromatography mass spectrometry (LCMS). Ang II levels were reduced by 83 ± 5 and 58 ± 4% over a 24-h period in the presence of ACE2 + 2A and ACE2 alone, respectively (*P*=0.008; Figure 1E). Consistent with this, Ang 1–7 levels increased in the presence of ACE2 + 2A by 165 ± 12% compared to ACE2 alone (*P*=0.003; Figure 1E) further supporting the notion that 2A stimulates ACE2 activity.

#### 2A can be detected in multiple organs when administered to normal mice

We next examined the tissue distribution of 2A (1 mg/kg) upon administration to normal wild-type C57BL/6J mice via intravenous (i.v), subcutaneous (s.c) or intranasal (i.n) routes. 2A was rapidly cleared from the circulation over the first 4h, which could reduce untoward off target effects of the peptide, however a small amount of peptide was still detected in plasma 24 h post administration (Figure 1F). Interestingly, irrespective of the route of administration, 2A was detectable at low concentrations in the heart, lung, spleen, kidney, brain and spinal cord 24-h post administration (Figure 1G).

We next examined the mechanism of action of 2A, and since ACE2 is the receptor for SARS-CoV-2^4^, we also examined how 2A affects the interaction between SARS-CoV-2 spike protein and ACE2.

### Molecular Docking predicts 2A weakens the binding between ACE2 and the SARS-CoV-2 spike protein

**Figure 2:**
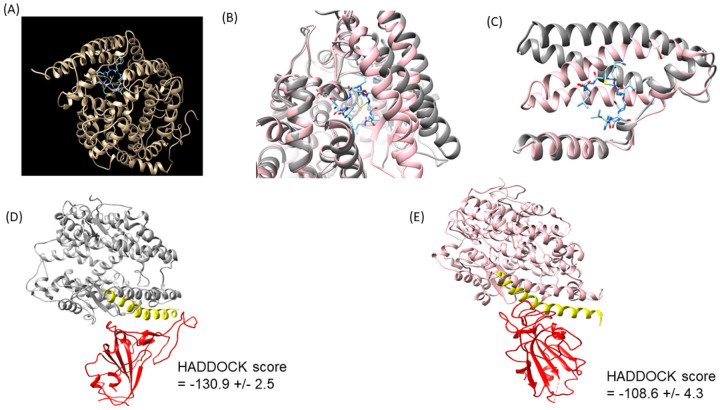
2A is predicted to weaken the interaction between SARS-CoV-2 and ACE2. PDB ID 1R4L was used as the template for the 2A and scrambled peptide docking analysis. (A)Ribbon representation of the peptide 2A/ACE2 receptor complex structure, (B)predicted 2A/ACE2 structure (shown in pink) docked into the published ACE2 pdb structure (pdb id: 1R42 shown in grey), (C) comparison of the conformation of the active sites of predicted and published ACE2 structures, Docking prediction of spike protein receptor binding domain (RBD) to ACE2 showing (D)top predicted pose for apo ACE2 (pdb id:7KJ2, shown in grey), and (E) 2A-modified ACE2 (shown in pink). The N-terminal RBD-binding helix of ACE2 shown in yellow. The spike protein RBD is shown in red. Abbreviations are as for Figure 1.

Molecular docking was employed to understand the mechanism of action of 2A. Binding studies indicate that 2A is buried deep inside the ACE2 structure (Figure 2A) and results in a clear change in conformation (Figure 2B). The two helices flanking the ACE2 active site (Glu22 - Leu91) change conformation to accommodate 2A (Figure 2C and supplementary figure 2A). Residues 22–53 comprise the binding site for the receptor binding domain (RBD) of the spike protein, and conformational changes here are likely to lead to disruptions in ACE2 binding. We propose that 2A binding induces a novel ACE2 conformation, distinct from other known structures, which may correspond to a SARS-CoV-2-resistant state.

Analysis of 2A-ACE2 interactions indicates that Lys7 in 2A forms a multitude of contacts with ACE2 (supplementary figure 2A – 2B), including hydrogen bonding with the backbone of Leu391, Asn394, and cation-p interactions with Phe40 and Phe390 (supplementary figure 2B). Leu10 in 2A also forms a hydrogen bond with Glu375. Alanine scanning mutagenesis studies indicate that residues 7 and 10 are critical for ACE2 stimulation. Hydrogen bond pairing plays an important role in the regulation of protein-ligand binding affinity^5^. In contrast, molecular docking of the control scrambled peptide suggests that it is unlikely to change ACE2 conformation (supplementary figure 2C). The scrambled peptide also forms fewer hydrogen bonds compared to 2A, with any given sidechain forming no more than one hydrogen bond with ACE2 (supplementary figure 2D).

Subsequent dockings of *apo* and 2A-modified ACE2 to the spike protein RBD using HADDOCK^6,7^ predict that *apo* ACE2 binds more strongly to the RBD (Figure 2D), with close resemblance to the ACE2-RBD crystal structure^8^, while the 2A-modified ACE2 binds more weakly (Figure 2E), with substantially fewer interactions. This result suggests that conformational changes induced by 2A leads to a weaker association between ACE2 and the virus, conferring resistance to infection by SARS-CoV-2. This may be particularly important for Variants of Concern (VOC) that more tightly bind to ACE2^9^. To corroborate our findings from molecular docking studies, we next examined whether 2A attenuates SARS-CoV-2 infection in primary human nasal epithelial cells. We selected these cells as ACE2 in primary human nasal epithelial cells plays a substantial role in SARS-CoV-2 infection.^10,11^

### Pre-treatment with 2A completely prevents SARS-CoV-2 infection in primary human nasal epithelial cells

**Figure 3:**
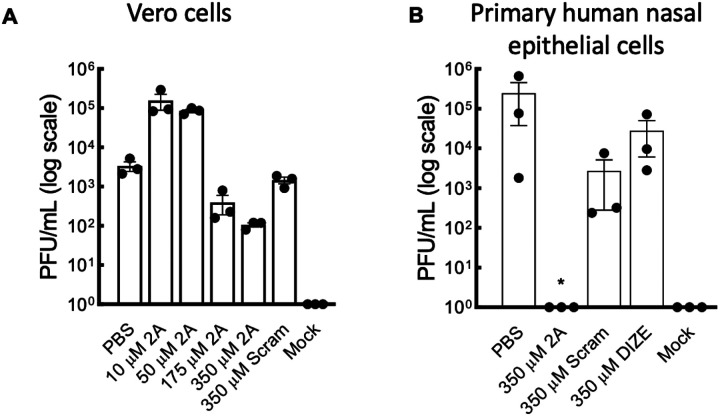
2A decreases the replication of SARS-CoV-2 *in vitro*. (A) Vero cells were treated with differing doses of peptide 2A, PBS or scrambled control peptide for 1 hour at 37 °C. Cells were then infected with SARS-CoV-2 (hCoV-19/Australia/QLD02/2020; EPI_ISL_407896; QLD02) at an MOI of 3 for 1 hour at 37 °C. Viral titres were assessed 16 hours post-infection. Each data point represents data from independent experiment (n=3), averaged from three technical replicates. (B) Primary nasal epithelial cells were differentiated at an air-liquid interface and then treated apically and basolaterally with PBS, peptide 2A, scrambled peptide or diminazene (DIZE) (350 μM). Cells were then infected with QLD02 at an MOI of 3 by adding virus to the apical layer for 1 hour at 37 °C. After infection, media in the apical layer was removed. Cells were then treated basolaterally with a dose of treatment (PBS, peptide 2A, scrambled peptide or DIZE; 350 μM) every 12-hours for a total of 48-hours at which time viral titres were assessed. Each data point represents a different donor (n=3). Normal distribution of data was assessed using a Shapiro-Wilk test. Data was analysed using a Kruskal-Wallis test with Dunn’s multiple comparison test relative to cells treated with PBS. *P<0.05. Mean ± SEM is shown.

To determine the appropriate dose of 2A to use in cell culture, a preliminary experiment was performed in post-infected Vero cells (Figure 3A). Sixteen hours post-infection cells treated with 350 μM of peptide 2A had the lowest titres of SARS-CoV-2 in cell culture supernatant although none of the 2A concentrations tested were significantly different from that of the PBS control. We therefore elected to use 350 μM of peptide 2A on primary human nasal epithelial cells (Figure 3B). To examine the potential of 2A as a prophylactic, we pre-treated the cells with 2A. Primary human nasal epithelial cells were differentiated at an air-liquid interface. Cells were then pre-treated apically and basolaterally with PBS, peptide 2A, scrambled peptide or DIZE for 1 h prior to infecting the cells with SARS-CoV-2. 2A, DIZE, PBS or scrambled peptide was added every 12 h for a period of 48 h. Viral titres produced by epithelial cells was subsequently assessed at 48 hours post-infection. Strikingly, no infectious virus was detected in the nasal epithelial cells treated with peptide 2A indicating that anti-viral effects of 2A can last up to 12 h. In contrast, viral titres in epithelial cells treated with scrambled peptide or DIZE were not significantly different to cells treated with PBS (Figure 3B). These findings in primary human nasal epithelial cells indicate 2A (350 μM) is effective in preventing infection when replaced at 12-h intervals and provide direct support for the vast potential of 2A as being used as a prophylactic. We next examined whether 2A can exert anti-inflammatory effects post-infection.

### Administration of 2A post SARS-CoV-2 infection improves the clinical score and lung inflammation in K18-hACE2 mice

**Figure 4:**
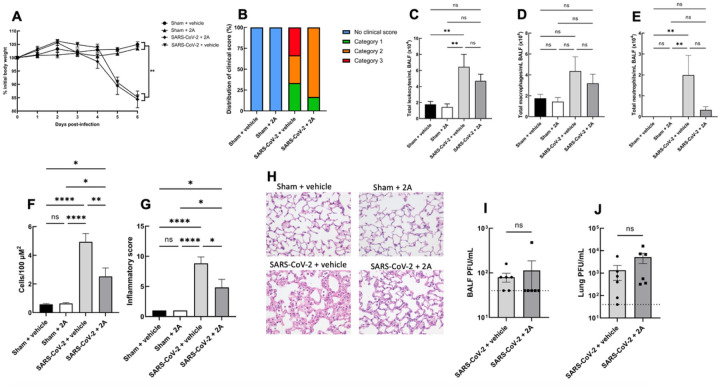
2A reduces clinical scores and lung inflammation in K18-hACE2 mice infected with SARS-CoV-2. (A) Body weights of K18-hACE2 mice infected with SARS-CoV-2 (or sham) and treated with 2A or vehicle from 3 days post-infection (n=6/group; two-way ANOVA). (B)Clinical scores of mice at 6 days post-infection, with category 3 representing severe and lethal disease presentation. (C) Total leukocyte counts in bronchoalveolar lavage fluid (BALF) at day 6 post-infection (one-way ANOVA). (D-E)Differential counts of leukocytes in BALF and stained with haematoxylin and eosin (H&E) (one-way ANOVA). (F) Total inflammatory cell counts from histological analysis of lung tissue. (G) Inflammatory scoring of lung architecture (one-way ANOVA). (H) Representative H&E images of lung architecture. Viral titres in (I) BALF, and (J) lung homogenates (unpaired *t*-test). *P<0.05, **P<0.01, ****P<0.0001. Data are mean ± SEM.

Data in primary human nasal epithelial cells provided a strong impetus for the further evaluation of 2A. We therefore examined the effect of 2A in a mouse model of SARS-CoV-2 infection. Murine ACE2 is incompatible with the spike protein of SARS-CoV-2.^12^ Transgenic mice which possess the human ACE2 receptor under the control of the K18 promoter (K18-hACE2 mice) are susceptible to infection.^12,13^ We infected K18-hACE2 mice with 10^3^ PFU of SARS-CoV-2 (Wuhan strain) or sham (PBS), and treated mice with 2A or vehicle from 3 days post-infection (dpi) (i.n; daily). Infected vehicle treated mice dramatically lost weight from 4 dpi, and developed severe clinical signs including laboured breathing, lethargy, hunching and ruffled fur requiring euthanasia (Figure 4 A–B). Infected mice treated with 2A (i.n) were protected against severe clinical signs with reductions in laboured breathing and lethargy. They also had fewer inflammatory cells in bronchoalveolar lavage fluid and in the airways under histological examination, as well as improved inflammatory scoring in the lung architecture (Figure 4 C–H). This provides evidence that 2A can improve lung inflammation when administered at 24-h intervals which is consistent with our tissue distribution data indicating 2A can be detected in lungs 24-h post intranasal delivery. Interestingly, we did not observe any significant differences in viral loads in bronchoalveolar lavage fluid or lung homogenates between mice treated with vehicle or 2A, suggesting a complex interplay between ACE2 stimulation and SARS-CoV-2 infection (Figure 4 I–J). Docking experiments predict 2A prevents infection by inducing a conformational change in ACE2 structure. Since we administered 2A to mice post-infection, it is not surprising that 2A did not reduce viral loads in these mice. In contrast, 2A was able to completely prevent viral infection in primary human nasal epithelial cells as 2A was administered pre-infection in this experiment.

Together, these findings indicate the vast potential of 2A as both a prophylactic and as anantiinflammatory drug post-infection. In this context, chronic inflammation together with altered ACE2 expression are hallmarks of diabetes which increase the risk of SARS-CoV2 infection.^14^ Therefore, we next examined the effects of 2A in type 1 diabetes.

### 2A treatment prevented the increase in blood glucose levels and loss of body weight in STZ-induced diabetic mice

Streptozotocin (STZ; 150 mg/kg; i.p) was used to induce type 1 diabetes in male C57BI/6J mice. Blood glucose was measured 1 week post STZ injection to confirm the presence of diabetes (BG levels > 15 mmol/L at 1 week post STZ injection was included in this study). After establishment of diabetes (T0), mice were randomly allocated to receive 2A (1 mg/kg/ day; 10% DMSO in saline) or vehicle (10% DMSO in saline) via subcutaneous minipumps for a period 3 months. Non fasting blood glucose levels were measured every 2 weeks until study end (T3), and body weight was measured daily, until study end. 24-h urine samples were collected at 1 month intervals since commencement of 2A or its vehicle. (Detailed Methods section is provided at the end of the Discussion).

**Figure 5:**
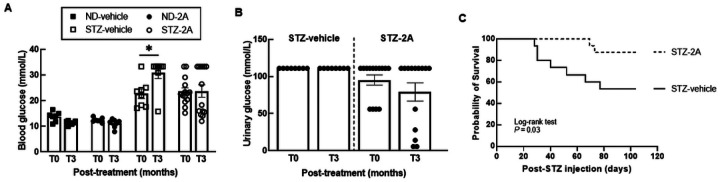
2A prevented the increase in blood glucose levels and loss of body weight in type 1 diabetic mice. (A) Blood glucose levels between pre-treatment (T0 months; mice have established diabetes but immediately prior to starting respective treatment) and post-treatment (T3 months; mice received treatment for 3 months) in ND-vehicle (*n* = 7), ND-2A (*n* = 7), STZ-vehicle (*n*= 8–15), and STZ-2A mice (*n* = 14–16). *P< 0.05, paired *t*-test. (B) Urinary glucose levels in STZ-vehicle and STZ-2A mice at T0 and T3 months posttreatment. Data are mean ± SEM. Abbreviations: ND: non-diabetic; STZ: streptozotocin. (C) Survival curve of STZ-vehicle, and STZ-2A mice over the 3 months treatment period. Number of animals in each group at the start of the experiment are as follows: ND-vehicle (*n* = 7; data not shown), ND-2A (*n* = 7; data not shown), STZ-vehicle (*n* = 15), and STZ-2A (*n* = 16). Each vertical drop indicates a death due to weight loss of ≥ 15% of the pre-diabetic body weight. All the non-diabetic mice survived until study end. Log-rank test was used to calculate the P-value of the survival curve, which indicate that there is a difference between the survival curves of the treatment groups (*P* = 0.03).

Blood glucose levels increased by 43 ± 16% (*P* = 0.03; paired t-test) at study end, compared to respective pre-treatment levels, in diabetic mice administered vehicle (Figure 5A; Supplementary Figure 3A). In contrast, blood glucose levels did not significantly change (P=0.93; paired t-test) in diabetic mice administered 2A (1 mg/kg/day; 3 months; s.c mini-pumps; Figure 5A; Supplementary Figure 3A). Urinary glucose excretion in diabetic mice receiving 2A or vehicle was not significantly different at study end (T3), compared to respective levels immediately prior to 2A or vehicle treatment (T0) (*P* ≥0.21; paired t-test;

Figure 5B). This suggests that 2A does not dampen the increase in blood glucose levels in diabetes via increasing its excretion.

Interestingly, 24-h urine volume at study end (T3) increased by 2 and 3 folds, respectively, in diabetic mice receiving vehicle or 2A, when compared with respective baseline levels immediately prior to administration of these respective treatments (T0; P <0.001; paired t-test; Supplementary Figure 3B). Further analysis indicate that 43% of diabetic mice receiving 2A had no change in 24-h urine volume from T0 to T3 (P = 0.52; paired t-test; Supplementary Figure 4A), with a parallel 28 ± 3% reduction in blood glucose content (P <0.001; paired t-test; Supplementary Figure 4B) and no significant change in urinary glucose excretion (P = 0.09; paired t-test; Supplementary Figure 4C). In 57% of diabetic mice administered 2A, 24-h urine volume increased by 3-fold from T0 to T3 (P <0.001; paired t-test; Supplementary Figure 4D). In this subset of mice, neither blood glucose nor urinary glucose changed from T0 to T3 (P ≥ 0.17; Supplementary Figure 4E - 3F). Of particular note, the cohort of diabetic mice administered 2A with a 3 fold increase in urine volume from T0 to T3, had 30% higher baseline (T0) blood glucose levels than the cohort of diabetic mice administered 2A with no change in urine volume from T0 to T3 (*P* = 0.02; unpaired t-test; Supplementary Figure 4B, 4E) suggesting that 2A is more effective in dampening the increase in blood glucose levels at early stages of diabetes.

47% of mice in the vehicle group were euthanized due to severe weight loss as per the ethics requirements. Only 12% of mice were culled in the cohort of diabetic mice receiving 2A (Figure 5C; Supplementary Figure 5A). Diabetic mice in the latter cohort were culled 60 days post-STZ injection or later, in the 90-day study protocol, while the majority of diabetic mice in the vehicle group were euthanized prior to 60 days post-STZ injection due to severe weight loss as per the ethics requirements (Figure 5C). Endpoint blood glucose in mice culled before study end are shown in supplementary figure 5B. Mice in non-diabetic groups survived until study end (data not shown).

2A had minimal effects on organ weight (Supplementary Table 1).

While the precise mechanisms by which 2A prevents the elevation in blood glucose levels and prevents loss of body weight remain to be determined, our observations nonetheless provide solid evidence that 2A has great promise in the treatment of type 1 diabetes. Protective arm of the RAS improves pancreatic beta cell function by improving the function of islet microvascular endothelial cells,^15^ a source which increasing evidence demonstrates is critical for the function of beta cells.^16^ In this context, IL-6 is a key inflammatory mediator in type 1 diabetes as well as in SARS-CoV2 related inflammation.^17–19,19,20^ Therefore, we next examined whether 2A can reduce RAS induced increase in IL-6 expression in endothelial cells.

## Anti-inflammatory effects of 2A:

**Figure 6:**
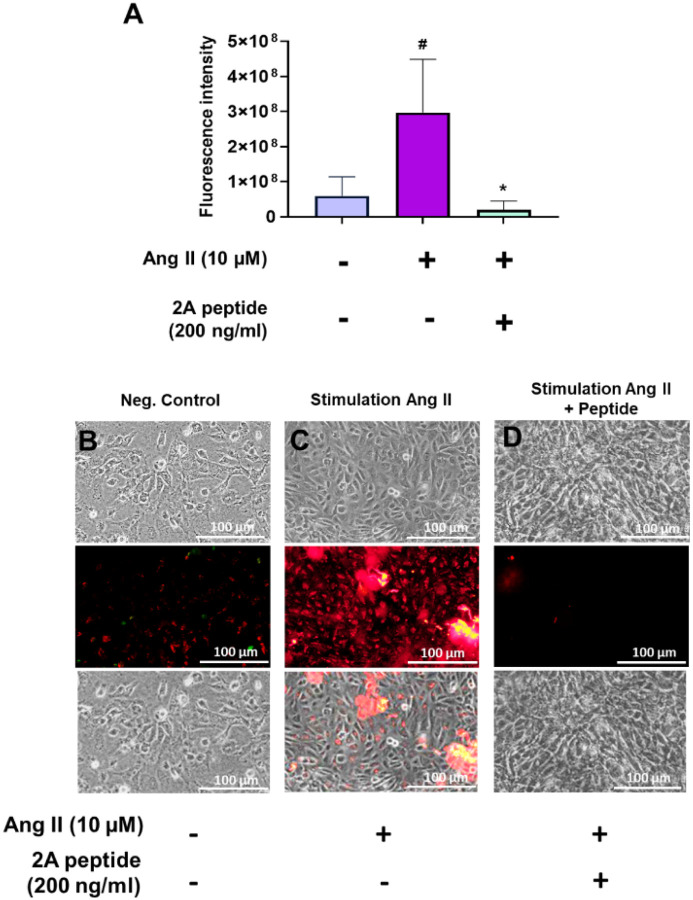
Measurement of IL (interleukin)-6 expression in SVEC-10 cells under physiological shear rate (1000 s^−1^). (A) Relative fluorescence intensity representing corresponding IL-6 expression in different groups. Values are mean ± SD, n=3, one-way ANOVA test. ^#^increased IL-6 expression compared to negative control: ^#^p< 0.05. *Reduced IL-6 expression compared to stimulant group: *p< 0.05. Representative brightfield, fluorescence and merged images of IL-6 expression in the negative control (B), stimulant (C), and peptide-treated (D) groups. The fluorescence signal was detected from the StarBright^™^ Blue 520 (red) tagged with a secondary antibody.

We used a straight channel microfluidic device mimicking the physiological microvascular environment to study the anti-inflammatory effects of 2A. IL-6 expression in cells lining the device was greater with AngII treatment compared with control. IL-6 expression was a near 14-fold less with AngII + 2A compared to Ang II alone. Near 3-fold reduction in IL-6 was also observed in the presence of Ang II and 2A compared with control (Figure 6A), suggesting decreased IL-6 expression and increased anti-inflammatory effect. Fluorescence images clearly showed the different levels of IL-6 expression in the three groups based on the red colour, and merged images proved the colocalization of IL-6 expression in the cells (Figure 6 C–D).

## Discussion

Here, we report the development of a novel drug lead ‘2A’ which stimulates ACE2 enzyme activity and has strong protective effects in SARS-CoV2 and type 1 diabetes.

2A provides a unique and first of its kind approach to simultaneously weaken the interaction of SARS-CoV-2 with ACE2 while also increasing the catalytic and hence the anti-inflammatory effects of ACE2. Our data indicate that 2A completely prevents SARS-CoV-2 infection in primary human nasal epithelial cells, and attenuates lung inflammation improving clinical presentation in transgenic K18-hACE2 mice infected with SARS-CoV-2. Consistent with this, molecular docking experiments predict that 2A reduces the binding affinity of SARS-CoV-2 spike protein to ACE2 by changing its conformation. These data are in line with previous findings indicating impaired ACE2 activity contributes to lung inflammation and disease severity.^13,18,21^ Of particular interest, 2A has strong clinical translational potential. Since nasal epithelial ACE2 plays a critical role in SARS-CoV-2 infection in humans,^11^ our observation that 2A can completely prevent SARS-CoV-2 infection indicates its vast potential in being used as a prophylactic. In addition, 2A reduces lung inflammation and improves clinical presentation in transgenic K18-hACE2 mice infected with SARS-CoV-2. While there are anti-inflammatory drugs which provide benefit to patients with COVID-19 to varying degrees, there is an unmet need to develop new therapeutic approaches to more effectively target lung inflammation in SARS-CoV-2,^22–25^ which contributes substantially to patient mortality. 2A represents a new class of anti-inflammatory drugs, which not only weakens the interaction between SAR-CoV-2 and ACE2 but also exerts anti-inflammatory effects via stimulating ACE2 enzyme activity.

We next examined the effects of 2A in type 1 diabetes, a pathological condition characterized by systemic inflammation and altered ACE2 expression resulting in increased risk of severe COVID-19. Reduced ACE2 expression is reported in patients with type 1 diabetes which contributes substantially to disease pathology.^15,26^ Our data indicate 2A completely prevents the increase in blood glucose levels in mice with STZ induced type 1 diabetes and ameliorates the related loss of body weight. 2A also completely prevents RAS induced IL-6 expression in endothelial cells in a microfluidic device mimicking the physiological environment, indicating its vast potential in exerting anti-inflammatory effects in the setting of SARS-CoV2 and type 1 diabetes.

We did not observe any untoward effects in our animal models suggesting 2A has strong clinical translation potential. Our observations provide a solid basis to develop a novel class of anti-inflammatory drugs with broad implications in both non-communicable and communicable diseases.

## Methods

### Discovery of 2A from a snake venom

We previously reported that a synthetic peptide corresponding to the first 20 amino acids of *Bothrops asper* myotoxin II increases the activity of several Zn^2+^ dependent metalloproteases including ACE-2^3^. We then made a peptide library (Genic Bio Ltd, Shanghai, China) by deleting two amino acids at a time from the C or N-termini, and both termini simultaneously. These peptides were screened using the QFS based ACE-2 assay described below. The selected peptide 2A was C-terminally amidated to improve stability.

Crude pepsets consisting of Alanine substituted analogues of 2A were synthesized (Genic Bio Ltd, Shanghai, China) for initial screening using the QFS based ACE-2 assay. Thereafter the Alanine substituted analogues of interest were synthesised at >95% purity for further testing.

### Effects of 2A on ACE2 catalytic activity

#### Measurement of recombinant human ACE2 enzyme activity using quenched fluorescence substrate-based assay.

All QFS-based assays were conducted in 96-well format. In each of the QFS-based assays described below, fluorescence was measured using λ_ex_ = 320 nm and λ_em_ = 405 nm at 37 °C. Enzymes were incubated with 2A or truncated peptides for 1 h at 37° C prior to adding appropriate QFS (40 μM) indicated in Table 1. The reaction rate was calculated from the linear portion of the fluorescence curve. Specific enzyme activity was calculated using a standard curve of known concentrations of 7-methoxycoumarin-4-acetic acid (fluorophore). All synthetic peptides were synthesised by GeniBio Ltd (Shanghai, China). All recombinant human enzymes were purchased from R&D systems (Minneapolis, USA).

#### The effects of truncated analogs of the parent peptide of 2A and increasing concentrations of amidated 2A on rhACE2 activity

Each truncated peptide (2.6μM) or 2A (0.9 – 26 μM) was incubated with rhACE2 (0.1 ng/μL) for 1 h at 37 °C before adding appropriate QFS to start the enzyme reaction. Fluorescence was measured as described above.

*The effects of amidated 2A on rhACE2 catalytic activity and the activity of its related enzymes* rhACE2 (0.1 ng/μL), rhIDE (0.1 ng/μL), rhNEP (0.05 ng/μL), rhECE-2 (0.05 ng/μL), and rhECE-1 (6.4 ng/μL) were incubated with 2A (2.6 μM) at 37 °C for 1 h before adding QFS. Enzyme control only had its respective buffer. Fluorescence was measured as described above.

#### The effects of amidated 2A on rhACE2 enzyme kinetics

rhACE2 (0.1 ng/μL) was incubated with 2A (2.6 μM) at 37 °C for 1 h. Reactions were started by adding QFS (0–100 μM final concentration). V_max_ and K_m_ were calculated using non-linear regression analysis (Michaelis-Menten equation in GraphPad prism software; version 8.2.0).

#### Effect of alanine scan analogues on rhACE2 activity

A library of 2A analogs were synthesised where one amino acid residue at a time was replaced by an Ala residue. These analogs were initially synthesised as crude pepsets and were screened for their effects on rhACE2 activity. From these pepsets, 7 analogs that induced an increase in ACE2 activity were identified and resynthesised at 95% purity. These 7 peptides (2.6 μM) were then re-screened for their effects on ACE2 activity using the QFS-based assay. Each peptide was incubated with rhACE2 at 37 °C for 1 h before adding QFS.

#### The effects of 2A on ACE2 dependent breakdown of angiotensin II and formation of angiotensin 1–7

The effect of 2A on ACE2-mediated cleavage of angiotensin II was assessed using LCMS. rhACE2 (0.1 ng/μL) was incubated with 2A (1.7 μM) for 1 h at 37 °C. Angiotensin II (0.02 μg/μL) was then added. Aliquots of equal volumes were collected at T = 0, 3, 6 and 24-h. Aliquots were immediately acidified with trifluoroacetic acid (0.1% final). Samples were snap frozen in dry ice and lyophilized for analysis by LCMS.

Prior to loading, samples were reconstituted in 100 μL loading buffer. Samples were analysed using a quadrupole TOF mass spectrometer (MicroTOFq, Bruker Daltonics, Bremen, Germany) coupled online with a 1200 series nano HPLC (Agilent Technologies). Samples were loaded onto a zorbax 300SB reversed-phase trap column equilibrated with 95% buffer A (0.1% formic acid). The flow rate was set to 10 μL/min. The components were eluted over a 10 min gradient to 70% buffer B (80% acetonitrile, 0.1% formic acid) and peptides were separated on zorbax 300SB-C18 nano column (75 μm × 15 cm, 3.5 μm). The eluant was nebulised and ionised using the Bruker nano-ESI source with a capillary voltage of 4500 V, dry gas at 180° C, flow rate set at 51 μL/min and nebuliser gas pressure at 300 mbar. The mass spectrometry acquisition was in selected ion monitoring mode after selected ion extraction of the mass spectrometry spectra. Data was processed using Skyline (version 2119.1.0.193, University of Washington; Washington, USA) to perform ion chromatogram extractions and peak integrations.

## Pharmacokinetics and tissue distribution of 2A *in vivo*

All experimental procedures involving animals were performed following approval from the animal ethics committee of the University of Queensland. Experimental procedures were conducted as per the National Health and Medical Research Council of Australia policies and guidelines for the care and use of animals for scientific purposes (8th Edition, 2013). Wild-type C57BL/6J mice (male, 10–12 weeks old) were purchased from the Animal Resources Centre (Western Australia, Australia). All animals were housed within the University of Queensland Biological Resources animal facility in a pathogen-free environment with a 12 h dark/12 h light cycle and free access to food and water.

The pharmacokinetic (PK) profile and biodistribution of 2A peptide was assessed in mice (n=5; per route at a dose of 1 mg/kg) after intravenous (i.v; tail vein), subcutaneous (s.c; between the shoulders) and intranasal (i.n; 10μl in each nostril with total volume of 20μl) administration. Blood samples were collected at 10, 20, 30, 45, 60 mins, 2 h and 4 h after drug administration via the tail vein using a microsampling technique^27^. At the terminal time point (24 h post administration), animals were anaesthetised with zolazepam (50 mg/kg) and xylazine (12 mg/kg) via i.p. injection. Blood samples were then collected via cardiac puncture as well as tail bleed to validate the sampling protocol. Plasma was isolated from blood samples by centrifugation (2000 g for 10 min at 4 °C), and stored at −80 °C. Following blood collection at the termination time point, mice were immediately perfused transcardially with saline to remove blood from tissues. Harvested tissue samples were then weighed and homogenized in an equal weight volume of milliQ water. On the day of analysis, aliquots (5 μL) of thawed plasma samples and 100μL of tissue homogenates were mixed with internal standard (10 μL) and processed for peptide quantification using a semi-validated LC–MS/MS method as described previously^28^.

Quantification of 2A peptide was performed using AcF-[OPdChaWR] peptide as an internal standard as described previously^28^. Briefly, samples were vortexed with 10 μL of IS followed by deproteinisation by 100 μL of ice-cold acetonitrile with 2% formic acid for plasma samples and 300 μL for tissue homogenates solution and kept at 25°C for 15 min. The resulting solution was vortexed and centrifuged at 14,000 rpm for 10 min at 4 °C. Supernatant was transferred to fresh tubes and dried using freeze dryer. The residual was mixed with 50 μL of 75% methanol in water vortexed for 10 min at 25 °C, centrifuged 14,000 rpm for 10 min at 25 °C and upper clear 40 μL of supernatant was transfer in to HPLC vial containing inserts for analysis using an API 3200 (AB SCIEX) triple quadrupole LC-MS/MS. Chromatographic separation was implemented using a Kinetex EVO C18 analytical column (100 × 2.1 mm, 100 Å, 5 μm, Phenomenex Inc., CA, USA) under binary gradient conditions using mobile phase A (0.1% formic acid in LC grade milliQ water pH 3) and mobile phase B (0.1% formic acid in 100% acetonitrile solvent) with a 350 μL/min flow rate. Concentrations of 2A peptide in samples was measured against known concentration of internal standard and peak area ratio of analyte to internal standard.

## Stability of 2A in cell culture

HEK293 (passage 24) were seeded into tissue-culture-grade 96-well plates at a density of 20,000 cells/well. After 24 h, cells were washed with PBS and replaced with Opti-MEM reduced serum media containing amidated or non-amidated 2A (9 μM). Aliquots of equal volume were taken at T = 0 and 60 min. Amidated 2A was added to control wells having media (no cells) only. Samples were immediately acidified in 0.1% TFA, snap frozen in dry ice and stored in −80 °C until analyzed by LCMS.

Samples were analyzed by LC-MS/MS on a Shimadzu Nexera uHPLC (Japan) coupled to a Triple Tof 5600 mass spectrometer (ABSCIEX, Canada) equipped with a duo electrospray ion source. One μl of each extract was injected onto a 2.1 mm × 100 mm Zorbax C18 1.8um column (Agilent) at 200 μL/min. Linear gradients of 1–50% solvent B over 7 min at 200 μL/min flow rate, followed by a steeper gradient from 50% to 98% solvent B in 1 min were used for peptide elution. Solvent B was held at 98% for 3 min for washing the column and returned to 1% solvent B for equilibration prior to the next sample injection. Solvent A consisted of 0.1% formic acid (aq) and solvent B contained 90/10 acetonitrile/ 0.1% formic acid (aq). The ionspray voltage was set to 5500 V, declustering potential (DP) 100 V, curtain gas flow 25, nebuliser gas 1 (GS1) 50, GS2 to 60, interface heater at 150 °C and the turbo heater to 500 °C. The mass spectrometer acquired 500ms full scan high resolution, at 30,000 resolving power, TOF-MS data over the mass range m/z 600 to 1200.

Both amidated and non amidated 2A were quantified using the [M+2H]^2^+ doubly charged molecular ion from m/z 575.32 to 575.37.

The data was acquired using Analyst TF 1.6 and quantification was carried out using MultiQuant 2.1.1 software (ABSCIEX, Canada).

## Studies in Vero Cells and Primary Human Nasal Epithelial Cells

### Virology assays

#### Cell collection and ethics statement

Primary nasal epithelial cells were collected from healthy adult (aged 25–34 donors by placing a sterile nasal mucosal curette (Arlington Scientific Inc., USA) in the mid-inferior portion of the inferior turbinate. Informed consent was obtained from all donors. This study was approved by the University of Queensland’s Human Research Ethics Committee (2017000520).

#### Cell culture

Vero E6 cells were purchased from ATCC (ATCC CRL-1586^™^) and were maintained in DMEM (Gibco, USA), containing 10% (v/v) heat-inactivated foetal bovine serum (Cytiva), 100 U/ml penicillin and streptomycin (Life Technologies, Australia).

Primary nasal epithelial cells were expanded and passaged in PneumaCult EX Plus media (STEMCELL Technologies Inc, Canada). After initial expansion, the nasal epithelial cells were seeded at a density of 3–5×10^5^ cells/transwell on 6.5 mm transwell polyester membranes with 0.4 μm pores (Corning Costar, USA) and cultured in PneumaCult EX Plus media (STEMCELL Technologies Inc, Canada). Cells were monitored for confluence. When a confluent monolayer was achieved, cells were ‘air-lifted’ by removing the media from the apical chamber and replacing the basolateral media with PneumaCult air liquid interface (ALI) media (STEMCELL Technologies Inc, Canada). Medium was replaced in the basal compartment three times a week, and the cells were maintained in ALI conditions for at least 3 weeks until ciliated cells and mucus were observed and cells obtained a transepithelial electrical resistance (TEER) measurement greater than 1000Ω. Differentiated cultures were then infected with SARS-CoV-2.

#### Viral stocks

SARS-CoV-2 isolate hCoV-19/Australia/QLD02/2020 was provided by Queensland Health Forensic & Scientific Services, Queensland Department of Health. Virus was grown on VeroTMPRSS2+ cells and titred^29^. All studies with SARS-CoV-2 were performed under physical containment 3 (PC3) conditions and were approved by the University of Queensland Biosafety Committee (IBC/374B/SCMB/2020).

#### Viral infection

Nasal epithelial cells at an air liquid interface were pre-treated apically and basolaterally with peptide 2A, DIZE, scrambled peptide (350 μM), or PBS for 1 hour at 37 °C. Apical media was then removed and cells were infected with mock (PBS) or SARS-CoV-2 at a multiplicity of infection (MOI) of 3. 100 μL of virus or PBS was placed in the apical compartment and incubated for 1 hour at 37 °C. Following incubation, excess virus was removed from the transwell and cells were incubated at 37 °C with 5% CO_2_. Every 12 hours, another dose of peptide 2A, DIZE, scrambled peptide or PBS was added to the basolateral compartment. At 48 hours post-infection, 100 μL of PBS was added to the apical compartment and cells were incubated for 10 mins at 37 °C with 5% CO_2_. The apical supernatant was subsequently removed and stored at −80 °C.

Vero cells were grown to confluence overnight and treated with peptide 2A, scrambled peptide, or PBS for 1 hour at 37 °C. Cells were infected with mock (PBS) or SARS-CoV-2 at a multiplicity of infection (MOI) of 3. 8 hours later, peptide 2A, scrambled peptide (350 μM) or PBS were added to the culture. 8 hours later (i.e. 16 hours post-infection) supernatant was collected for plaque forming assays.

#### Viral Plaque Forming Assay

SARS-CoV-2 in supernatants were quantified by plaque-forming assays in Vero cells as described by Gordon et. al^29^. Cells were seeded in 6-well plates at 0.3 × 10^6^ cells per well. The next day, the cells were infected by ten-fold serial dilutions of SARS-CoV-2 samples in serum-free minimum essential media (MEM; Gibco, USA) for 1 hour at 37 °C, rocking every 10 minutes. After infection, overlay medium (MEM supplemented with 2% FCS and 0.05% agarose) was added to the wells. Assays were incubated at 37 °C for 65 hours before fixation in 4% formalin (Sigma-Aldrich, USA) and visualization using 0.025% crystal violet (Sigma-Aldrich, USA) in 20% ethanol solution.

## Studies in K18-hACE2 mice

### SARS-CoV-2 infection of K18-hACE2 mice

SARS-CoV-2 mouse infection experiments were approved by the SLHD Institutional Biosafety Committee and Animal Ethics Committee. Hemizygous female K18-hACE2 mice (Tg(K18-ACE2)2Prlmn/J) were bred in-house at the Centenary Institute and maintained under specific pathogen-free conditions. Mice at approximately 8 weeks of age were transported to our PC3/BSL3 facility for SARS-CoV-2 challenge. Mice were anaesthetized with isoflurane followed by intranasal challenge with 10^3^ PFU SARS-CoV-2 (VIC01/2020) in a 30 μL volume. After infection, mice were housed in the IsoCage N biocontainment system (Tecniplast, Italy) and given access to standard rodent chow and water *ad libitum*. Mice were weighed and monitored daily, with increased monitoring when mice began to develop symptoms. Mice commenced daily intranasal treatments with 30 μL of 2A (1 mg/kg in 10% DMSO/PBS) or vehicle (10% DMSO/PBS) from 3 days post-infection. Clinical scoring of mice was conducted prior to euthanasia, with mice categorised based on: Cat 1 weight loss, early lethargy and hunching; Cat 2 substantial weight loss, lethargy, hunching, ruffling and early laboured breathing, and Cat 3 based on exacerbations of Cat 2 signs accompanied by complete immobility. At day 6 post-infection, mice were euthanised by intraperitoneal overdose with pentobarbitone (Virbac, Australia). Blood was collected via heart bleed, and serum collected (10,000 × g, 10 minutes) at room temperature. Multi-lobe lungs were tied off and bronchoalveolar lavage fluid collected from the single lobe lung in 1 mL HANKS solution (Sigma-Aldrich, USA) using a blunted 19G needled inserted into the trachea. Bronchoalveolar lavage fluid was centrifuged (300 × g, 7 minutes) and the supernatant collected and placed at −80 °C for further analysis. The cell pellet was resuspended in 200 μL of Red Blood Cell Lysis Buffer (ThermoFisher, Australia) and incubated for 5 minutes, followed by addition of 700 μL of HANKS solution and centrifuged as previously outlined. Following centrifugation, the supernatant was discarded and the cell pellet was resuspended in 160 μL of HANKS solution. Total cell counts were enumerated using a disposable haemocytometer (Sigma-Aldrich, USA). Differential counts were performed by loading the cell suspension into a disposable cytospin funnel and pelleting cells (300 × g, 7 minutes) onto glass slides. Cytospin slides were then stained using QuickDip Stain Kit (Modified Giemsa Stain) protocol as per manufacturer’s instructions (POCD Scientific, Australia) and cells differentiated using an inverted light microscope. Multi-lobe lungs were collected and either frozen and stored at −80 °C for further analysis, or homogenised in 2 mL HANKS solution using a GentleMACS tissue homogeniser. Lung homogenate was centrifuged (300 × g, 7 min) to pellet cells, followed by collection of supernatants for plaque assays. The single lobe lung was perfused with 0.9% NaCl_2_ via the heart, followed by inflation through the trachea with 0.5 mL 10% neutral buffered formalin and then submerged in formalin solution. Following 2 week fixation, lungs were transported to a PC2 facility where they were paraffin-embedded, cut to 3 μm thick sections using a Leica microtome (Leica, Germany), and then stained using Quick Dip Stain Kit. Inflammatory cells in the single lobe lung sections were enumerated using a Zeiss Axio Imager.Z2 microscope with a 40X objective (Zeiss, Germany). Inflammatory scoring was applied to lung architecture based on an unbiased scoring system encompassing inflammation in the parenchyma, vasculature and the airways, with 3 being the lowest score possible representing little or no inflammation, while a score of 13 was attributed to the highest inflammatory score representing severe and excessive inflammation.

#### Docking study methods

The HADDOCK webserver was used to predict the binding of the two conformations of ACE2 to the spike protein RBD. The 2A-free ACE2 and the spike protein RBD structures employed for HADDOCK calculations were those obtained from PDBID: 7KJ2^30^. We utilized the MDockPep program^5^ to perform the peptideprotein docking simulation, which is accessible at https://zougrouptoolkit.missouri.edu/mdockpep/. MDockPep is a state-of-the-art method for predicting the protein-peptide complex structures and is developed for addressing the challenge of predicting all-atom structures of protein-peptide complexes without any prior knowledge about the peptide binding site and the bound peptide conformation. It provides significantly better performance better than existing docking methods. In benchmarking tests, it successfully generated near-native peptide binding modes in 95.0% of the bound docking cases and in 92.2% of the unbound docking cases^5^. The 2A-modified ACE2 structure employed was that predicted using MDockPep. The active residues for both ACE2 conformations were specified as residues 27–38, while those for the spike protein RBD were specified as residues 489–496. Default values for all other parameters were used. Schroedinger Maestro (18) was used to produce 2D ligand-receptor interaction diagrams.

#### Ethics approval

AH animal experiments were approved by the Animal Ethics Committee, University of Queensland. Experimental procedures were conducted as per the National Health and Medical Research Council of Australia policies and guidelines for the care and use of animals for scientific purposes (8th Edition, 2013). All animals were housed within the University of Queensland Biological Resources animal facility in a pathogen-free environment and in a controlled environment (temperature: 22–26 °C; humidity: 40–60%; 12 h light-dark cycle) with free access to food and water.

#### Model of type 1 diabetes

*Streptozotocin (STZ) is a widely used model of type I diabetes which closely mimic the human disease^31,32^. Male C57Bl/6J mice were purchased from Animal Resources Centre (Western Australia, Australia) and these mice were acclimatized for at least one week prior to inducing diabetes. At 20–22 weeks of age, mice were injected with a single high dose of STZ (150 mg/kg; intraperitoneal) in 0.1 M sodium citrate buffer (pH 4.5) to induce diabetes or with 0.1 M sodium citrate alone as non-diabetic control. Mouse body weight was measured daily. Blood glucose levels were measured via tail bleed 1-week post-STZ injection to confirm the establishment of diabetes (CareSens N; iSENS, Seoul, Korea). Animals with blood glucose levels of >15 mmol/L at 1-week post-STZ injection were included in this study. Blood glucose levels were measured every 2 weeks until the end of the treatment period. Mice were placed in metabolic cages to collect 24-h urine samples. Mice were then randomly allocated to one of the following groups (n = 7–14 per group):* STZ-2A, STZ-vehicle, non-diabetic (ND)-2A, ND-vehicle. Mice were treated with 2A (1 mg/kg/day; 10% DMSO in saline) or vehicle (10% DMSO in saline) for 12 weeks via subcutaneous osmotic mini-pumps. Osmotic mini-pump surgery was conducted under isoflurane anaesthesia (4% induction, 1–2% maintenance). Osmotic mini-pumps were replaced every 4 weeks while mice were under isoflurane anaesthesia. After 4, 8 and 12 weeks of 2A or vehicle treatment, mice were placed in metabolic cages for 24-h urine collection. Mice were euthanized in a CO_2_ chamber at the end of the treatment period.

#### Measurement of urinary glucose levels

Keto-Diastix Reagent Strips (Ascensia Diabetes Care, Basel, Switzerland) were used to measure urinary glucose levels. Urine samples were thawed at room temperature prior to the assay. Measurements were made as per the Glucose Colour Chart provided by the manufacturer.

#### Statistical analysis

All data are presented as mean ± SEM. Statistical analyses were performed on GraphPad Prism software (Version 9.0.2) by unpaired t-test for dichotomous comparisons, and one-way ANOVA followed by Tukey’s post-hoc test for multiple comparisons. Paired t-test was performed to compare within the group. Log-rank (Mantel-Cox) test was conducted for statistical analysis of the survival curve. *P*< 0.05 was considered statistically significant.

## Assessing anti-inflammatory effects of 2A peptide under physiological shear rate

To test the anti-inflammatory effect of 2A peptide at the physiological shear rate, we used a simple straight channel PDMS (Polydimethylsiloxane) microfluidic device. The device micmicked blood vessel where the channel was coated with endothelial monolayer. The dimension of the channel is 20 mmx 100 μm x50 μm (lengthx widthx height).

### Cell culture

In this study, we used SVEC4–10, a murine endothelial cell line; from ATCC, Rockville, MD, which was cultured in DMEM (Dulbecco’s Modified Eagle Medium) high glucose medium supplemented with 100 IU/ml of penicillin G, 100 μg/ml streptomycin, and 10% (vol/vol) FBS (fetal bovine serum). Cells were cultured in an incubator with 5% CO_2_ at 37 C and subcultured when they reached 90% confluence, and passage-6 was used in this experiment.

### Coating and growing SVCE-10 cells in PDMS device under flow conditions

The device was washed with 70% ethanol, sterile PBS (pH-7.4) and treated under UV for an hour to sterilize completely. The PDMS device was coated with bovine skin collagen (Sigma-Aldrich). Briefly, a 10 μL of collagen stock (3 mg/mL) was added to 90 μL of high glucose DMEM to get the final working concentration at 300 μg/mL. A 20 μL working solution was infused through the device and incubated for an hour in the cell incubator (5% CO_2_, 37°C). The solution was taken out and the device was washed with PBS. The device was placed in the cell incubator at 37 C for 15 min for drying. In the meantime, the SVEC-10 cells were trypsinized and detached from the flask. The cells were collected and resuspended in high glucose DMEM supplemented with FBS and antibiotics. A 10 μL of cell suspension (10 million cells/mL) was infused through the channel. The device was placed in a shaker and incubated at 37°C and 200 rpm for an hour. Then, the device was transferred to the cell incubator and incubated for another two hours. Next, the device was placed upside down and incubated for two hours to ensure cell attachment in the device. After five hours of incubation, the device was connected to a peristaltic pump, complete DMEM media was perfused at a flow rate of 12 μL/min (~400 s^−1^), and cells were allowed to continue growing under flow condition overnight. Then, the flow rate was increased to 31 μL/min, corresponding to the physiological shear rate 1000 s^−1^ and ran for 24 h to reach the confluent SVEC-10 monolayer. The flow rate was calculated using Newton’s law of viscosity^33^.


(1) $$\tau \;=\;\eta \;x\;\frac{dv}{dx}$$

(2) $$\tau \;=\;\;\frac{\text{6}\eta \text{Q}}{h^{2}w}$$

Where, τ = shear stress, η= dynamic viscosity (Pa.s), $\frac{dv}{dx}$ = shear rate (s^−1^), Q= flow rate, h=device’s height, w=device’s width. Also, η for cell culture media is considered as equivalent to water = *8.90 × 10^−4^ pa·s*.

### Evaluating anti inflammatory effects of 2A peptide

After reaching the confluent monolayer of SVEC-10 in the device at 1000 s^−1^, the cells were treated with Angiotensin II (10 μM) for 24 h at the same shear rate to induce inflammation. This group was considered as a positive control or stimulant group. To detect the anti-inflammatory effects of the 2A peptide, the cells were treated with 2A peptide (200 ng/ml) along with Angiotensin II (10 μM) 1000 s^−1^ for 24 h. Negative control was prepared by perfusing complete cell media without any other addition at 1000 s^−1^ for 24 h. The cells were then fixed by 4% PFA for 15 min under the same flow rate at room temperature for immunostaining. The cells were washed with PBS for 5 min under flow condition and stored at 4°C before use. The cells were incubated with IL (interleukin)-6 primary antibody for 24 h at 4°C, washed thrice with PBS and then incubated for an hour with secondary antibody (Goat ANTI-RABBIT IgG StarBright^™^ Blue 520) at room temperature. Finally, the device was washed with PBS thrice for imaging. Brightfield and fluorescence images were taken using an inverted microscope (CKX53, Olympus) mounted with a digital camera (DP74, Olympus) and operated by CellSens Software Version 3.1. Data (*n*=3) was analyzed by image processing software FiJi/ImageJ (Java 1.8.0_172).

## Data analysis

### 2A Discovery

Enzyme activity and kinetic parameters were compared using an unpaired Student’s t-test or one-way ANOVA followed by Tukey post-hoc test. Statistical analysis was conducted using GraphPad prism software (version 8.0.2).

### Stability and tissue distribution of 2A in vivo

LC-MS/MS data processing and analysis were performed using Analyst software (AB SCIEX, Applied Biosystems Inc., USA, version 1.5.1) and Multiquant software (AB SCIEX, USA, version 2.0). Pharmacokinetics data analysis was performed using Pharsight Phoenix WINNONLIN software using non-compartmental analysis (Certara, L.P, St. Louis, Missouri, USA, v8.1). The linear trapezoidal rule was applied for the calculation of area under the curve (AUC 0-t) of the plasma concentration vs time profiles.

### Studies in Vero Cells and Primary Human Nasal Epithelial Cells

Results from viral plaque forming assay was compared against PBS using Kruskal-Wallis test and multiple comparisons test. Outliers were removed by ROUT with *P*<0.05.

### Studies in KT8-hACE2 mice

Body weight curves were analysed using a two-way ANOVA. BALF, differential counts, inflammatory cell counts and inflammatory scoring were analysed using a one-way ANOVA. Viral titres were analysed using unpaired Student’s *t*-tests. Data shown is the mean ± SEM. *P<0.05, **P<0.01, ****P<0.0001. All data were analysed using GraphPad Prism version 9.0.

## Figures and Tables

**Table 1.** The buffer compositions and respective quenched fluorescent substrate used for each enzyme.

Enzyme	Buffer	Quenched fluorescence substrate
rhNEP	50 mM TrisHCl, 150 mM NaCI; pH 6.3	(7-methoxycoumarin-4-yl)acetyl-Arg-Pro-Pro-Gly-Phe-Ser-Ala-Phe-Lys-(2,4-dinitrophenyl)-OH
rhIDE	50 mM TrisHCl, 1 M NaCl; pH 7.5	
rhECE-1	50 mM TrisHCl, 150 mM NaCl; pH 6.3	
rhECE-2	100 mM MES, 250 mM NaCl; pH 5.75	
rhACE-1	50 mM HEPES, 300 mM NaCl; pH 8.3	
rhACE2	100 mM Tris-HCl, 1 M NaCl; pH 6.5	(7-methoxycomarin-4-yl)acetyl-Ala-Pro-Lys-(2,4-dinitrophenyl)-OH

rhNEP: recombinant human neprilysin, rhIDE: recombinant human insulin degrading enzyme, rhECE-1: recombinant human endothelin converting enzyme-1, rhECE-2: recombinant human endothelin converting enzyme-2, rh-ACE-1: recombinant human angiotensin converting enzyme-1, rhACE2: recombinant human angiotensin converting enzyme 2.

## Data Availability

All data are available in the main text or the supplementary materials.
